# Availability of Cost-effectiveness Studies for Drugs With High Medicare Part D Expenditures

**DOI:** 10.1001/jamanetworkopen.2021.13969

**Published:** 2021-06-18

**Authors:** Rebecca L. Tisdale, Iris Ma, Daniel Vail, Jay Bhattacharya, Jeremy D. Goldhaber-Fiebert, Paul A. Heidenreich, Alexander T. Sandhu

**Affiliations:** 1 Health Services Research and Development, Center for Implementation to Innovation, Veterans Affairs Palo Alto Health Care System, Menlo Park, California; 2 Stanford Health Policy, Centers for Health Policy/Primary Care and Outcomes Research, Department of Medicine, Stanford University School of Medicine and the Freeman Spogli Institute for International Studies, Stanford, California; 3 California Pacific Medical Center, San Francisco; 4 Department of Surgery, Stanford University, Stanford, California; 5 Veterans Affairs Palo Alto Health Care System, Palo Alto, California; 6 Division of Cardiology, Department of Medicine, Stanford University School of Medicine, Stanford, California

## Abstract

**Importance:**

Prescription drug spending in the US requires policy intervention to control costs and improve the value obtained from pharmaceutical spending. One such intervention is to apply cost-effectiveness evidence to decisions regarding drug coverage and pricing, but this intervention depends on the existence of such evidence to guide decisions.

**Objective:**

To characterize the availability and quality of cost-effectiveness studies for prescription drugs with the greatest Medicare Part D spending.

**Design, Setting, and Participants:**

In this national cross-sectional analysis, publicly available 2016 Medicare drug spending records were merged with 2016 US Food & Drug Administration Orange Book data and the Tufts Medical Center Cost-Effectiveness Analysis (CEA) Registry. All studies published through 2015 that evaluated the cost-effectiveness of the 250 drugs for which Medicare Part D spending was the greatest in US-based adult patient populations were included. Data were analyzed from September 2018 to June 2020.

**Main Outcomes and Measures:**

The presence and quality of published cost-effectiveness analyses for the 250 drugs for which Medicare Part D spending was greatest in 2016 were assessed based on the inclusion of key cost-effectiveness analysis elements and global ratings by independent reviewers for the Tufts CEA Registry.

**Results:**

Medicare Part D spending on the 250 drugs in the sample totaled $122.8 billion in 2016 (84.1% of total spending). Of these 250 drugs, 91 (36.4%) had a generic equivalent and 159 (63.6%) retained some patent exclusivity. There were 280 unique cost-effectiveness analyses for these drugs, representing data on 135 (54.0%) of the 250 drugs included and 67.0% of Part D spending on the top 250 drugs. The 115 drugs (46.0%) without cost-effectiveness studies accounted for 33.0% of Part D spending on the top 250 drugs. Of the 280 available studies, 128 (45.7%) were industry sponsored. A large proportion of the studies (250 [89.3%]) did not meet the minimum quality requirements.

**Conclusions and Relevance:**

In this cross-sectional study, a substantial proportion of 2016 Medicare Part D spending was for drugs with absent or low-quality cost-effectiveness analyses. The lack of quality analyses may present a challenge in efforts to develop policies addressing drug spending in terms of value.

## Key Points

**Question:**

What are the availability and quality of cost-effectiveness studies on drugs with the greatest Medicare Part D spending?

**Findings:**

In this cross-sectional study of 250 drugs with the greatest Medicare Part D spending in 2016, cost-effectiveness analyses were unavailable for 46.0%, with these drugs representing 33.0% of Medicare Part D spending. For the 54.0% of drugs with available cost-effectiveness studies, many of the studies did not meet minimum quality standards.

**Meaning:**

This study showed that a substantial proportion of 2016 Medicare Part D spending was for drugs with absent or low-quality cost-effectiveness analyses, which may present a challenge in efforts to develop policies addressing drug spending in terms of value.

## Teaser

This cross-sectional study examines the availability and quality of published cost-effectiveness studies for drugs with the greatest Medicare Part D spending in 2016.

## Introduction

Before the COVID-19 pandemic, analysts forecasted that the US would spend $350 billion^1^ on prescription drugs in 2020 (nearly 10% of US health care spending^2^), whereas the global pharmaceutical market would increase to $1.3 trillion worldwide.^1^ Although pandemic-related systemic shocks may alter these figures, there remains a consensus that the current situation is unsustainable and requires policy intervention.^3^ One potential intervention is a value-based drug formulary design, which seeks to price drugs based on their value for patients.^4^

A value-based drug formulary design incorporates the cost-effectiveness of a drug when determining coverage or cost-sharing for the consumer.^5^ A cost-effectiveness analysis (CEA) compares marginal benefits and marginal costs of treatment with the next-best alternative. Data from CEAs have formed the basis for health care reimbursement schemes in many developed countries other than the US; for example, the National Institute for Health and Care Excellence has used CEAs to guide the National Health Service in the UK.^6^ In principle, data from CEAs can be used to promote access to high-value therapies, encourage additional development of high-value treatments, and provide information regarding reducing excess spending on low-value treatments.

In the US, Medicare Part D is the prescription drug program for more than 40 million Medicare beneficiaries.^7^ With more than $100 billion of annual expenditures, it is responsible for more than 30% of all prescription drug spending in the US.^8^ Although certain drugs, specifically those administered in physician offices or infusion centers, are paid for by Medicare Part B insurance, Medicare Part D spending composes 77% of total Medicare drug spending.^9^ A value-based drug formulary design depends on data from CEAs to guide decisions. However, few data exist regarding the extent to which the cost-effectiveness of the drugs responsible for the bulk of US pharmaceutical spending is studied. Therefore, we evaluated the availability and quality of published CEAs on the drugs for which Medicare Part D spending was the greatest in 2016.

## Methods

### Data

In this cross-sectional study, we identified relevant drugs using the 2016 Medicare Part D Prescriber Public Use File.^10^ We chose to study the 250 drugs for which Medicare Part D spending was greatest because this subset represented 84.1% of total drug spending. The institutional review board of Stanford University deemed this study exempt from review and approval and the need for informed consent because it did not meet the definition of human subjects research and the data were deidentified. The study followed the Strengthening the Reporting of Observational Studies in Epidemiology (STROBE) reporting guideline.^11^

We evaluated drug characteristics using the 2016 US Food & Drug Administration (FDA) Orange Book,^12^ the FDA’s source for information regarding therapeutic equivalence, approval dates, and exclusivity. We merged drug data with the Tufts Medical Center Cost-Effectiveness Analysis Registry,^13^ a comprehensive registry of CEAs reporting results in dollars per quality-adjusted life-year (QALY) gained. The database includes detailed information regarding CEAs, including each study’s assessed quality as rated by an independent reviewer. We identified relevant cost-effectiveness studies published through 2015 to identify available studies at the start of 2016. The registry version used in the present study was updated through 2015 and thus included the most recent data at the time of our database query.

### Outcomes

From the FDA Orange Book and the Medicare Part D Prescriber Public Use File, we captured multiple drug characteristics. These included the year of initial FDA approval of the main ingredient, the type of application, exclusivity status and whether a broad or exact generic equivalent existed in 2016, the number of Medicare Part D 30-day standardized fills, the aggregate cost paid for Medicare Part D claims, and out-of-pocket spending by Medicare Part D beneficiaries.

The Tufts Medical Center Cost-Effectiveness Analysis Registry includes multiple study characteristics. These include whether the study was industry sponsored; whether a time horizon was stated and, if so, its magnitude; the analytic perspective (eg, societal, health care payer, or other) as reported by the authors and assessed by the reviewers; whether costs and QALYs were discounted and at what rate(s); what cost-effectiveness thresholds were used, including upper and lower bounds; the type of sensitivity analyses tested (bounded, probabilistic, univariate, or multivariate); whether a cost-effectiveness acceptability curve was included; and an overall quality rating for the study. To collect these data, each article was independently reviewed by 2 readers with training in decision analysis and CEA; after their review, the 2 readers convened for a consensus audit to resolve any potential discrepancies, and a third reader resolved disputed items.^14^

The First and Second Panels on Cost-effectiveness in Health and Medicine have made specific recommendations regarding how analyses should approach each of these study characteristics.^15,16^ On the basis of these recommendations, we considered studies to have the recommended time horizons if the time horizon was either lifetime or at least 30 years; the recommended perspectives if they took either a societal or health care payer perspective; and the recommended discounting if both costs and QALYs were discounted and at the same rate. Although there is no single recommended cost-effectiveness threshold, we considered studies to have a recommended cost-effectiveness threshold if the widely used threshold of $50 000 per QALY gained was used. In a sensitivity analysis, we evaluated whether each study used a threshold of either $50 000 or $100 000 per QALY gained. All parameters were taken directly from the registry.

For CEA sensitivity analyses, we evaluated multiple criteria: the inclusion of either univariable or multivariable sensitivity analyses, the inclusion of a probabilistic sensitivity analysis, and the inclusion of a cost-effectiveness acceptability curve. In addition and distinct from the other criteria evaluated, each study received a cumulative quality rating between 1 (lowest quality) and 7 (highest quality) by the registry reviewers. According to the registry, scores should reflect the following considerations: (1) whether incremental cost-effectiveness ratios were correctly computed, (2) comprehensive characterization of uncertainty (ie, probabilistic or nonprobabilistic evaluation of how changes in plausibly important assumptions affected the results), (3) correct treatment and explicit specification of health economic assumptions (discount rate, currency, and analysis time horizon), and (4) appropriate and explicit estimation of utility weights (with the importance of this item depending on the extent to which the intervention affected morbidity and mortality).^14^ We considered these quality metrics to represent the minimum criteria that CEAs should fulfill. Therefore, we assumed that an intermediate- to high-quality study would have a rating of 5 or higher.

### Statistical Analysis

The drugs identified from the Medicare Part D Prescriber Public Use File and their generic equivalents from the Orange Book were assigned to their most representative disease areas by 2 of us (I.M., A.T.S.) (eTable 1 in the Supplement), and another 1 of us (R.L.T.) acted as a third evaluator and adjudicator. To link the drugs to relevant CEAs, 3 of us (R.L.T., I.M., A.T.S.) compiled a list of pertinent search terms for each drug (eTable 2 in the Supplement). With these lists of search terms, we queried the Tufts database using Python, version 3.7.4 (Python Software Foundation) for comparative effectiveness studies to check whether the title or abstract mentioned the drug of interest.

After the initial automated screen, 2 of us (A.T.S. and R.L.T.) manually assessed the remaining pharmaceutical studies of US-based populations in the Tufts database for inclusion. We excluded studies in pediatric populations, studies that did not include a comparison arm without the drug of interest, and studies that focused on a screening strategy for selecting drug treatment (eg, genetic screening followed by selection of a drug). Additional details about the exclusion criteria are provided in eTable 3 in the Supplement. Data were collected for a single year, 2016, during which a large proportion of spending was for new antiviral drugs for hepatitis C, especially ledipasvir-sofosbuvir. As a sensitivity analysis, we repeated the analysis with exclusion of the new hepatitis C antiviral drugs (daclatasvir, elbasvir-grazoprevir, ledipasvir-sofosbuvir, sofosbuvir, and sofosbuvir-velpatasvir).

We then compiled descriptive statistics on the included drugs, studies, and drug-study pairs using Excel, version 16.48 (Microsoft Corporation); Stata, version 16 (StataCorp LLC); and Python, version 3.7.4. We separately analyzed branded drugs with no generic equivalent. We tested differences using *t* tests and χ^2^ tests, as applicable, with a 2-sided *P* < .05 considered statistically significant. The data were analyzed from September 2018 to June 2020.

## Results

### Drug Characteristics

**Figure 1.**
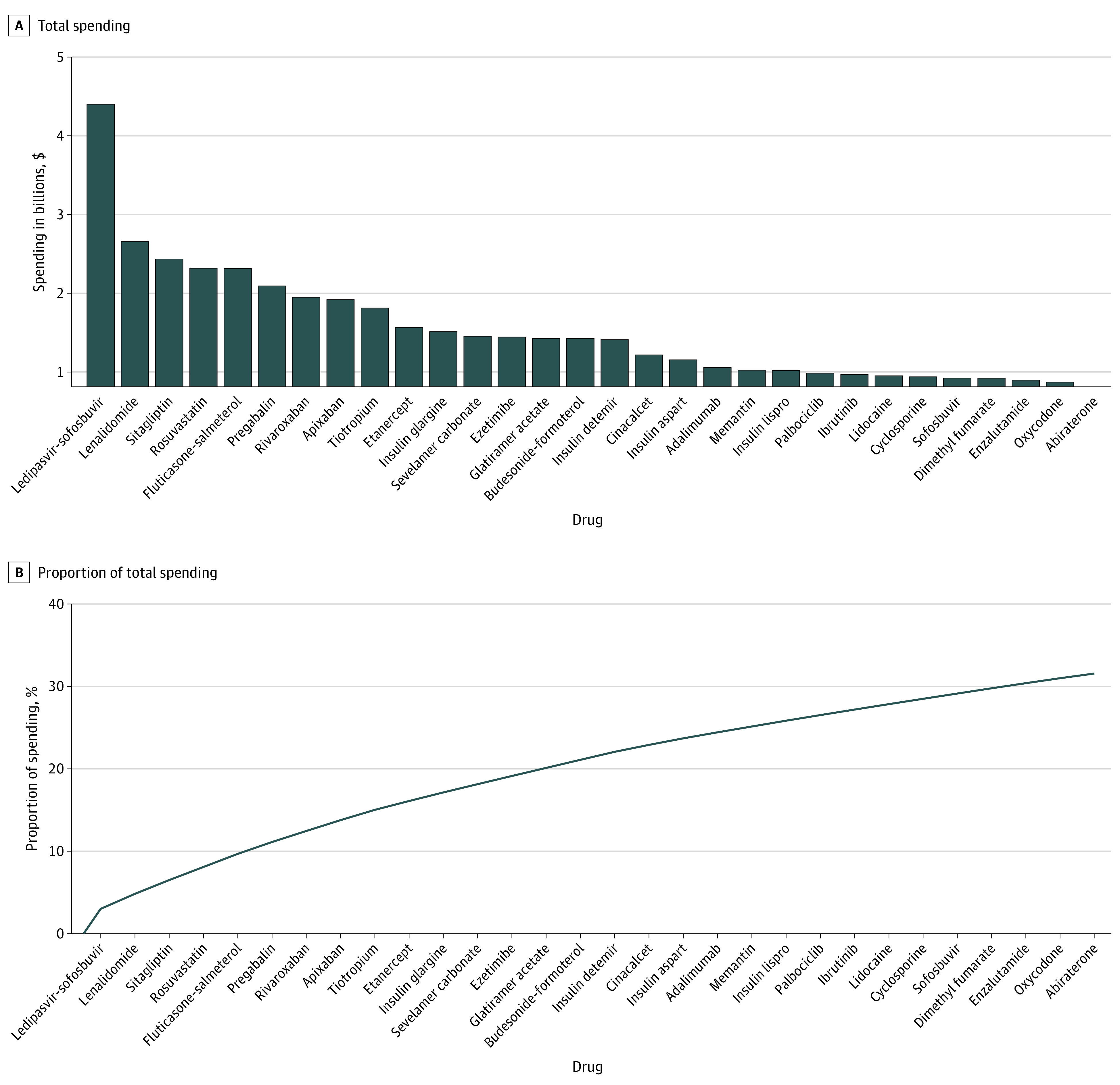
Top 30 Medicare Part D Drugs by Spending in 2016

Total Medicare Part D spending on drugs in 2016 was $146.1 billion; as shown in Figure 1 and Table 1, spending was highly concentrated. The top 30 drugs accounted for 33.5% of total Part D spending ($48.9 billion), and the top 250 drugs accounted for 84.1% of overall spending ($122.8 billion). Of these 250 drugs, 91 (36.4%) had a generic equivalent, whereas the remaining 159 (63.6%) retained some exclusivity.

**Table 1.** Characteristics of the 250 Drugs With the Greatest Medicare Part D Spending in 2016

Drug type	All drugs, No.	Generic drugs, No.	Median year of approval	2016	
				Prescriptions, No.^a^	Total spending, millions USD
All drugs	250	91	2004	1 567 760	122 754
By disease area					
Allergy	4	0	2003	2125	766
Cardiology	38	23	2000	699 579	17 536
Dermatology	3	2	1984	3328	585
Endocrinology	27	3	2007	195 643	23 334
Gastroenterology	11	5	2006	106 338	5136
Gynecology	2	1	1984	7442	656
Hematology	4	0	2004	1036	782
Hepatology	8	1	2014	1285	7069
Infectious diseases	19	4	2006	7758	4862
Nephrology	2	1	1996	30 215	2030
Neurology	23	10	2007	51 653	10 974
Oncology	21	3	2011	7402	11 596
Ophthalmology	10	5	2005	27 638	3087
Pain	12	12	1994	125 266	6090
Psychiatry	20	12	2004	147 273	7194
Pulmonology	22	1	2007	75 084	11 999
Rheumatology	15	2	2005	23 531	6469
Urology	9	6	2001	55 163	2590

^a^ Thousands of standardized 30-day fills.

Disease areas with the highest spending were endocrinology ($23.3 billion), cardiology ($17.5 billion), and pulmonology ($12.0 billion). Disease areas with the most prescriptions for standardized 30-day fills were cardiology (approximately 700 million), endocrinology (approximately 196 million), and psychiatry (approximately 147 million).

### Cost-effectiveness Studies

As shown in the eFigure in the Supplement, we excluded 5453 of 5769 cost-effectiveness studies in the Tufts database because they did not reference any of the top 250 drugs by spending or they did not analyze a US adult population. We excluded another 38 studies in which an alternate intervention rather than the specific drug was evaluated or in which the drug of interest was included in all study arms. Ultimately, we included 280 unique studies in the sample.

Results regarding the availability of cost-effectiveness evidence are shown in Table 2. Of the 250 drugs for which Medicare Part D spending was the greatest, 135 (54.0%) were included in at least 1 cost-effectiveness study. The 280 unique studies included in the cost-effectiveness studies data set represented 402 drug-study pairs (ie, many studies involved >1 of the drugs of interest). Drugs with any cost-effectiveness evidence represented 67.0% of the total spending among the top 250 drugs. Drugs without cost-effectiveness evidence represented 33.0% of spending among the top 250 drugs.

**Table 2.** Availability of Economic Studies on the 250 Drugs With the Greatest Medicare Part D Spending in 2016

Drug type	Economic studies available			Economic studies not available	
	Drugs, No.	Studies, No.^a^	Spending, %^b^	Drugs, No.	Spending, %^b^
Total	135	402	67.0	115	33.0
Patented	78	223	50.0	81	24.1
Generics	57	176	17.0	34	8.9
Approval year					
Before 2010	120	355	53.9	77	24.1
2010 or Later	15	47	13.0	38	8.9
Disease area					
Allergy	0	0	0.0	4	0.6
Cardiology	24	105	10.7	14	3.6
Dermatology	2	2	0.4	1	0.1
Endocrinology	11	34	13.2	16	5.8
Gastroenterology	6	15	1.6	5	2.5
Gynecology	2	6	0.5	0	0.0
Hematology	3	11	0.4	2	0.3
Hepatology	6	17	5.3	2	0.5
Infectious diseases	12	38	2.7	7	1.3
Nephrology	1	1	1.2	1	0.5
Neurology	14	34	7.2	10	1.7
Oncology	12	35	6.2	9	3.3
Ophthalmology	5	6	1.5	5	1.0
Pain	8	18	3.7	4	1.3
Psychiatry	13	39	4.6	7	1.3
Pulmonology	6	9	3.1	16	6.7
Rheumatology	9	25	4.3	6	1.0
Urology	3	7	0.4	6	1.7

^a^ There were 280 unique studies, but data represent drug-study pairs.

^b^ Denominator is total Medicare Part D spending on the 250 drugs with the greatest spending in 2016.

The 250 drugs with the greatest spending included 159 drugs with exclusivity, which accounted for 74.1% of total spending among these 250 drugs. No relevant published CEA was found for 81 (50.9%) of the 159 drugs with exclusivity. There was no difference in the quality of studies for drugs approved before vs after 2010.

The characteristics of the included studies are summarized in Table 3. Of the 280 unique studies, 128 (45.7%) were industry sponsored. Of all cost-effectiveness studies, 140 (50.0%) used an appropriate time horizon. Altogether, 96 studies (34.3%) had neither univariate nor multivariate sensitivity analyses, and only 136 (48.6%) of the studies had both. Only 159 (56.8%) of the studies included probabilistic sensitivity analyses. Only 144 (51.4%) of the studies included the recommended cost-effectiveness threshold of $50 000 per QALY gained. In a sensitivity analysis, 175 (62.5%) of the studies included cost-effectiveness thresholds of $50 000 and/or $100 000 per QALY gained.

**Table 3.** Characteristics of Economic Studies for the 250 Drugs with the Greatest Medicare Part D Spending in 2016

Study type	Total studies, No.	Rating, median (IQR)	Recommended study feature, No. (%)							
			Time horizon	Perspective	Discounting	Cost-effectiveness threshold	Univariate and multivariate sensitivity analyses	Univariate or multivariate sensitivity analyses	Probabilistic sensitivity analysis	Cost-effectiveness acceptability curve
All	280	5.0 (4.0-5.5)	140 (50.0)	264 (94.3)	256 (91.4)	144 (51.4)	136 (48.6)	184 (65.7)	159 (56.8)	98 (35.0)
By disease area^a^										
Allergy	0	NA	NA	NA	NA	NA	NA	NA	NA	NA
Cardiology	70	5.3 (4.5-5.5)	41 (58.6)	65 (92.9)	64 (91.4)	40 (57.1)	33 (47.1)	45 (64.3)	39 (55.7)	27 (38.6)
Dermatology	2	3.5 (3.3-3.8)	0	2 (100)	1 (50.0)	1 (50.0)	0	0	0	0
Endocrinology	25	5.0 (3.5-5.5)	19 (76.0)	24 (96.0)	22 (88.0)	12 (48.0)	8 (32.0)	15 (60.0)	12 (48.0)	10 (40.0)
Gastroenterology	13	4.5 (4.5-5.0)	4 (30.8)	12 (92.3)	11 (84.6)	4 (30.8)	4 (30.8)	8 (61.5)	7 (53.8)	1 (7.7)
Gynecology	6	4.5 (4.5-4.9)	0	6 (100)	6 (100)	2 (33.3)	2 (33.3)	3 (50.0)	3 (50.0)	0
Hematology	11	4.5 (3.5-5.3)	4 (36.4)	9 (81.8)	9 (81.8)	6 (54.5)	6 (54.5)	8 (72.7)	7 (63.6)	3 (27.3)
Hepatology	12	5.5 (4.9-6.0)	11 (91.7)	12 (100)	12 (100)	7 (58.3)	5 (41.7)	5 (41.7)	5 (41.7)	4 (33.3)
Infectious diseases	29	5.0 (4.0-5.5)	20 (69.0)	29 (100)	28 (96.6)	16 (55.2)	15 (51.7)	22 (75.9)	16 (55.2)	8 (27.6)
Nephrology	1	6.0 (6.0-6.0)	0	1 (100)	1 (100)	1 (100)	1 (100)	1 (100)	1 (100)	1 (100)
Neurology	24	4.5 (3.5-5.6)	5 (20.8)	22 (91.7)	24 (100)	12 (50.0)	14 (58.3)	17 (70.8)	15 (62.5)	9 (37.5)
Oncology	29	5.0 (4.5-5.5)	15 (51.7)	26 (89.7)	26 (89.7)	16 (55.2)	16 (55.2)	19 (65.5)	18 (62.1)	11 (37.9)
Ophthalmology	3	4.5 (4.0-5.3)	0	3 (100)	2 (66.7)	2 (66.7)	0	1 (33.3)	0	0
Pain	14	4.3 (4.0-5.6)	3 (21.4)	13 (92.9)	13 (92.9)	7 (50.0)	5 (35.7)	7 (50.0)	7 (50.0)	3 (21.4)
Psychiatry	27	4.5 (4.0-5.8)	5 (18.5)	26 (96.3)	26 (96.3)	13 (48.1)	18 (66.7)	11 (77.8)	11 (77.8)	14 (51.9)
Pulmonology	7	5.0 (4.3-5.0)	1 (14.3)	7 (100)	6 (85.7)	5 (71.4)	5 (71.4)	6 (85.7)	5 (71.4)	3 (42.9)
Rheumatology	16	5.3 (4.9-5.6)	7 (43.8)	15 (93.8)	15 (93.8)	5 (31.3)	9 (56.3)	12 (75.0)	10 (62.5)	9 (56.3)
Urology	7	4.5 (4.0-5.0)	6 (85.7)	7 (100)	6 (85.7)	5 (71.4)	5 (71.4)	7 (100)	6 (85.7)	2 (28.6)

Abbreviations: IQR, interquartile range; NA, not applicable.

^a^ Column does not sum to overall number of studies because some studies may have included drugs assigned to multiple disease categories and thus may be counted more than once.

Of the 135 drugs included in a CEA, 130 (96.3%) were included in at least 1 study that used a societal or health care payer perspective. The median study quality rating score was 5. Only 57 studies (20.4%) had a score of 6 or higher, whereas 80 (28.6%) had a score of 4 or lower. Of the 135 drugs included in cost-effectiveness studies, 37 (27.4%) were not included in studies with a quality rating of 5 or higher, and 81 (60.0%) were not included in studies of quality 6 or higher. After restricting analyses to the higher-rated studies, only 54.7% and 31.5% of total Medicare Part D spending on the top 250 drugs was for drugs included in CEAs with quality ratings of 5 or 6, respectively. Overall, 250 drugs (89.3%) did not meet all quality metrics, 30 studies (10.7%) met all quality metrics, and only 26 studies (9.3%) met all metrics with an overall quality rating of 5 or higher.

Study characteristics stratified by disease category are also shown in Table 3. There was substantial variation in study quality across the different categories. For multiple categories, the median quality score was lower than 5.

**Figure 2.**
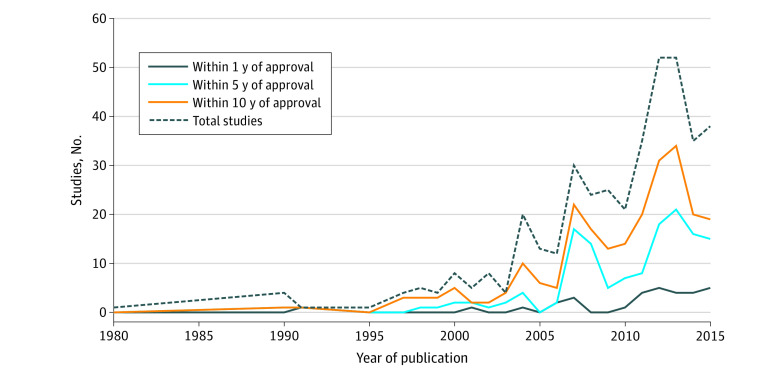
Trends in Publications of Cost-effectiveness Analyses for Drugs Covered by Medicare Part D

Trends in the time from drug approval to publication of CEAs are depicted in Figure 2. A total of 134 studies (47.9%) relevant to drugs in this study were published within 5 years before 2016, and 212 (75.7%) were published within 10 years before 2016. Examining the 402 drug-study pairs, few studies (31 [7.7%]) were published within 1 year of FDA approval; a larger proportion of studies (204 [50.7%]) were published from 1 to 10 years after drug approval. The overall number of published CEA studies for the drugs that we examined increased over time; 27 studies were published between 1980 and 2000, compared with 149 studies between 2010 and 2015.

We performed an analysis to evaluate whether the results were sensitive to the introduction of hepatitis C antiviral drugs in late 2014 and 2015. After removing all 5 hepatitis C drugs (totaling $6.3 billion in spending) from the original sample, there were 273 relevant CEAs compared with 280 in the larger sample. The main results were consistent: drugs with a CEA accounted for 65.7% of spending in the sample with the hepatitis C antiviral drugs removed compared with 67.0% in the sample with these drugs included.

## Discussion

This cross-sectional study assessed the availability and quality of published CEAs on drug treatment, which are important for implementation of value-based drug formulary programs. Despite liberal criteria for inclusion of studies in the sample, we found that 33.0% (>$40 billion) of Medicare Part D spending for the 250 drugs with the greatest spending in 2016 involved drugs without published CEAs. The gaps between existing cost-effectiveness evidence and Medicare spending were even larger after restricting the sample to drugs with high-quality cost-effectiveness studies whether based on a set of minimum quality criteria for CEAs or a global rating system. This paucity of data represents a challenge for efforts to evaluate drug purchasing in terms of value. Drug prices have been reported to be too high, access to therapies has been inconsistent, and neither pricing nor access has been considered to align with the clinical benefit or value for some drugs.^17^ Value-based approaches to formulary design and pricing are meant to correct this situation.

However, implementation of value-based approaches requires measurement of value. Cost-effectiveness analysis is a well-established approach for comparing clinical value across alternative treatments. A large number of developed countries other than the US constrain their drug prices using cost-effectiveness approaches that align cost with value; the UK and Australia are notable examples, with other countries benchmarking their own prices to those of the UK.^18^ In a value-based system, CEAs can provide a starting point for price or access negotiations. In addition, value-based formularies offer a potential solution to concerns regarding drug access.^19^ Minimizing cost sharing for high-value therapies may ensure improved access for therapies that are desirable and may promote broader access.^19^ Without peer-reviewed cost-effectiveness studies evaluating the cost and clinical benefit of a given drug, value-based drug formulary programs must rely on internal evaluations lacking this quality criterion.^20^

The results of the present study showed that cost-effectiveness studies were rarely published early enough to inform initial pricing. This finding implies that the cost-effectiveness of a given drug outside the manufacturer’s own internal economic analyses were less likely to be considered in initial pricing strategies. The Institute for Clinical and Economic Review and others have sponsored efforts to promote earlier analyses of the economic value of new drugs.^21^ The success of these endeavors near the time of market introduction will be critical for models that aim to use these analyses to inform drug pricing or access.

Quality varied among the published CEAs included in the present study. Most of the analyses adopted study perspectives and incorporated discounting according to the guidance of the First and Second Panels on Cost-effectiveness in Health and Medicine.^15,16^ However, we found low adherence to recommendations regarding the time horizon, cost-effectiveness threshold, or sensitivity analyses. These are minimum quality criteria that should be met by most studies. Performance of CEAs that use standard analytic approaches is necessary to compare different therapies. Increasing the quantity and quality of drug CEAs is important for the successful implementation of value-based formulary design.

Even when a study is well conducted based on the criteria used for quality assessment, the selection of parameter values, model structures, or outcomes to optimize the probability of a favorable evaluation of a given drug remains possible, which may lead to bias in industry-sponsored studies. Thus, the results of the present study should be viewed as representing the availability of CEAs that meet a minimum quality standard and an upper bound for availability of this evidence.

### Limitations

This study has limitations. First, we limited the sample to Medicare Part D drug spending for feasibility. Drugs administered in a clinic or inpatient setting are not covered by Medicare Part D. Thus, drugs and disease areas with disproportionate administration in these settings (eg, ophthalmology and oncology) are underrepresented in this sample. Therefore, these results may not fully capture the cost-effectiveness of these drugs and disease areas. Drug-specialty linkages are also subject to imprecision because many drugs have numerous indications across multiple specialties. Thus, linking drugs to the most relevant disease area can be somewhat arbitrary. For example, tumor necrosis factor α inhibitors are used frequently for various autoimmune diseases and could be categorized as primarily rheumatologic or gastrointestinal drugs given their use for inflammatory bowel disease.

We were also unable to categorize cost-effectiveness data by indication. Therefore, a drug may have been included in a cost-effectiveness study in our analysis but may have lacked a study focusing on its most common indications. We aggregated drugs with different formulations and indications given the challenge of accounting for this variation. We thus believe that our analysis represents an upper bound of the presence of relevant cost-effectiveness studies.

Conversely, some pertinent studies may have been excluded. For example, given that efficacy and cost can vary within a class of drugs, we did not view a study evaluating 1 proton pump inhibitor’s cost-effectiveness as applicable to other proton pump inhibitors. Likewise, although we used both an automated first screen and a subsequent manual search process to include all relevant studies, we may have inadvertently excluded relevant studies. Also, although we believe the Tufts Medical Center Cost-Effectiveness Analysis Registry is relatively comprehensive, there may be additional published economic analyses.

In addition, we analyzed Medicare Part D spending on drugs in a single year (2016). Events during calendar years may differ. One example is the introduction of new hepatitis C antiviral drugs (eg, ledipasvir-sofosbuvir) in late 2014 and 2015, which accounted for a substantial proportion of 2016 spending. Although results of our study were relatively unchanged after excluding those antiviral drugs in a sensitivity analysis, there may have been remaining idiosyncrasies in 2016 that could limit the generalizability of the findings.

## Conclusions

In this cross-sectional study, a substantial proportion of 2016 Medicare Part D spending was for drugs with absent or low-quality cost-effectiveness analyses. In addition, the quality of cost-effectiveness evidence was often inadequate. Improving the value of spending on prescription drugs may be considered an element of US health policy reforms in the future, and efforts for value-based reforms may be hampered by a lack of cost-effectiveness data.

## Notes

Supplement. **eFigure. **Flowchart **eTable 1.** Drug-disease area mapping **eTable 2.** Search terms **eTable 3.** Reasons for exclusion from database
