# Supplementary material for: Availability of Cost-effectiveness Studies for Drugs With High Medicare Part D Expenditures
Source: JAMA Netw Open. 2021 Jun 18;4(6):e2113969. doi: 10.1001/jamanetworkopen.2021.13969 (PMC8214163; doi:10.1001/jamanetworkopen.2021.13969)
Supplement: Supplement. — eFigure. Flowchart eTable 1. Drug-disease area mapping eTable 2. Search terms eTable 3. Reasons for exclusion from database [file jamanetwopen-e2113969-s001.pdf]

Supplementary Online Content

Tisdale RL, Ma I, Vail D, et al. Availability of cost-effectiveness studies for drugs with high Medicare Part D expenditures. *JAMA Netw Open*. 2021;4(6):e2113969. doi:10.1001/jamanetworkopen.2021.13969

eFigure. Flowchart
eTable 1. Drug-disease area mapping
eTable 2. Search terms
eTable 3. Reasons for exclusion from database

This supplementary material has been provided by the authors to give readers additional information about their work.

© 2021 Tisdale RL et al. *JAMA Network Open*.

Supplemental Appendices

eFigure. Flowchart

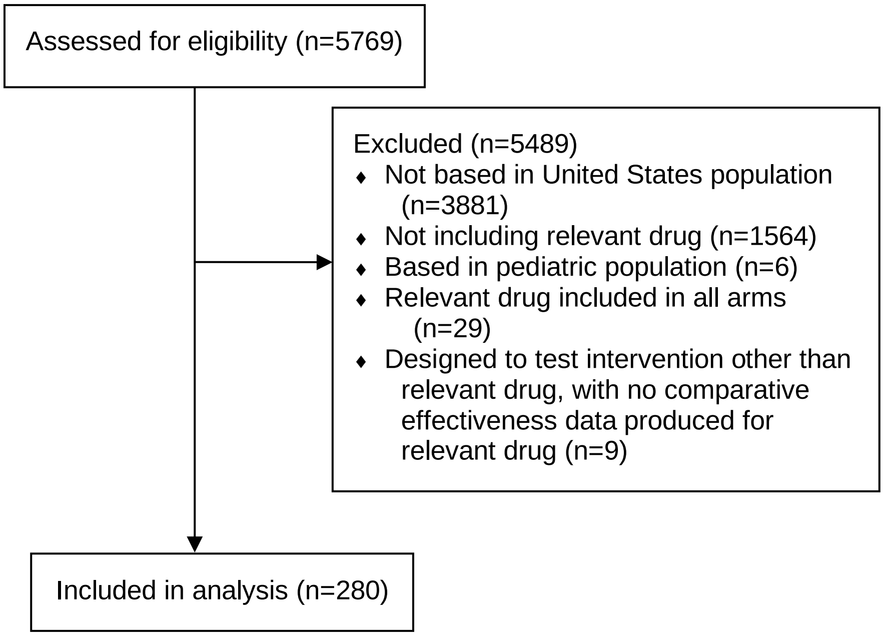

© 2021 Tisdale RL et al. *JAMA Network Open*.

eTable 1. Drug-Disease Area Mapping

**Drug**	**Disease Area**
C1 esterase inhibitor	Allergy
Epinephrine	Allergy
Icatibant	Allergy
Mometasone	Allergy
Alirocumab	Cardiovascular
Amlodipine	Cardiovascular
Amlodipine/olmesartan	Cardiovascular
Apixaban	Cardiovascular
Atorvastatin	Cardiovascular
Carvedilol	Cardiovascular
Clopidogrel	Cardiovascular
Colesevelam	Cardiovascular
Digoxin	Cardiovascular
Diltiazem	Cardiovascular
Dofetilide	Cardiovascular
Dronedarone	Cardiovascular
Enalapril	Cardiovascular
Ezetimibe	Cardiovascular
Ezetimibe/simvastatin	Cardiovascular
Fenofibrate	Cardiovascular
Furosemide	Cardiovascular
Hydrochlorothiazide	Cardiovascular
Hydrochlorothiazide/olmesartan	Cardiovascular
Isosorbide mononitrate	Cardiovascular
Lisinopril	Cardiovascular
Losartan	Cardiovascular
Metoprolol succinate	Cardiovascular
Metoprolol tartrate	Cardiovascular
Nebivolol	Cardiovascular
Niacin	Cardiovascular
Olmesartan	Cardiovascular
Omega-3	Cardiovascular
Prasugrel	Cardiovascular
Pravastatin	Cardiovascular
Ranolazine	Cardiovascular
Rivaroxaban	Cardiovascular
Rosuvastatin	Cardiovascular
Simvastatin	Cardiovascular
Ticagrelor	Cardiovascular

© 2021 Tisdale RL et al. *JAMA Network Open*.

Valsartan	Cardiovascular
Warfarin	Cardiovascular
Dabigatran	Cardiovascular
Clobetasol	Dermatology
Collagenase	Dermatology
Fluocinonide	Dermatology
Aspart	Endocrine
Aspart protamine	Endocrine
Canagliflozin	Endocrine
Cinacalcet	Endocrine
Corticotropin	Endocrine
Dapagliflozin	Endocrine
Degludec	Endocrine
Denosumab	Endocrine
Detemir	Endocrine
Dulaglutide	Endocrine
Empagliflozin	Endocrine
Exenatide	Endocrine
Glargine	Endocrine
Human insulin	Endocrine
Human insulin isophane (70/30)	Endocrine
Human insulin isophane	Endocrine
Levothyroxine	Endocrine
Linagliptin	Endocrine
Liraglutide	Endocrine
Lispro	Endocrine
Lispro protamine	Endocrine
Metformin	Endocrine
Metformin/sitagliptin	Endocrine
Saxagliptin	Endocrine
Sitagliptin	Endocrine
Teriparatide	Endocrine
Testosterone	Endocrine
Budesonide	Gastroenterology
Dexlansoprazole	Gastroenterology
Esomeprazole	Gastroenterology
Linaclotide	Gastroenterology
Lubiprostone	Gastroenterology
Mesalamine	Gastroenterology
Omeprazole	Gastroenterology
Pancrelipase	Gastroenterology
Pantoprazole	Gastroenterology

© 2021 Tisdale RL et al. *JAMA Network Open*.

Ranitidine	Gastroenterology
Teduglutide	Gastroenterology
Estradiol	Gynecology
Estrogen	Gynecology
Deferasirox	Hematology
Eltrombopag	Hematology
Enoxaparin	Hematology
Epoetin	Hematology
Daclatasvir	Hepatology
Elbasvir/grazoprevir	Hepatology
Ledipasvir/sofosbuvir	Hepatology
Rifaximin	Hepatology
Sofosbuvir	Hepatology
Sofosbuvir-velpatasvir	Hepatology
Tenofovir disoproxil	Hepatology
Ursodiol	Hepatology
Abacavir/dolutegravir/lamivudine	Infectious disease
Abacavir/lamivudine	Infectious disease
Atazanavir	Infectious disease
Cobicistat/darunavir	Infectious disease
Cobicistat/elvitegravir/emtricitabine/tenofovir alafenamide	Infectious disease
Daptomycin	Infectious disease
Darunavir	Infectious disease
Dolutegravir	Infectious disease
Doxycycline	Infectious disease
Efavirenz/emtricitabine/tenofovir	Infectious disease
Elvitegravir/cobicistat/emtricitabine/tenofovir disoproxil	Infectious disease
Emtricitabine/rilpivirine hydochloride/tenofovir disoproxil	Infectious disease
Emtricitabine/tenofovir	Infectious disease
Etravirine	Infectious disease
Raltegravir	Infectious disease
Ritonavir	Infectious disease
Valganciclovir	Infectious disease
Vancomycin	Infectious disease
Zoster vaccine	Infectious disease
Potassium chloride	Nephrology
Sevelamer	Nephrology
Aspirin-dipyridamole	Neurology
Baclofen	Neurology
Chlorpromazine	Neurology
Dalfampridine	Neurology
Dextromethorphan/quinidine	Neurology

© 2021 Tisdale RL et al. *JAMA Network Open*.

Dimethyl fumarate	Neurology
Divalproex	Neurology
Donepezil	Neurology
Droxidopa	Neurology
Fingolimod	Neurology
Glatiromer	Neurology
Interferon beta-1a	Neurology
Interferon beta-1b	Neurology
Lacosamide	Neurology
Levetiracetam	Neurology
Memantine	Neurology
Pregabalin	Neurology
Rasagiline	Neurology
Rivastigmine	Neurology
Sodium oxybate	Neurology
Teriflunomide	Neurology
Tetrabenazine	Neurology
Tizanidine	Neurology
Abiraterone	Oncology
Crizotinib	Oncology
Dasatanib	Oncology
Enzalutamide	Oncology
Erlotinib	Oncology
Everolimus	Oncology
Ibrutinib	Oncology
Imatinib	Oncology
Ixazomib	Oncology
Lenalidomide	Oncology
Methotrexate	Oncology
Nilotinib	Oncology
Nintedanib	Oncology
Osimertinib	Oncology
Palbociclib	Oncology
Pazopanib	Oncology
Pomalidomide	Oncology
Raloxifene	Oncology
Ruxolitinib	Oncology
Sorafenib	Oncology
Sunitinib	Oncology
Bimatoprost	Ophthalmology
Brimonidine	Ophthalmology
Brimonidine/timolol	Ophthalmology

© 2021 Tisdale RL et al. *JAMA Network Open*.

Brinzolamide	Ophthalmology
Cyclosporine	Ophthalmology
Difluprednate	Ophthalmology
Latanoprost	Ophthalmology
Nepafenac	Ophthalmology
Olopatadine	Ophthalmology
Travoprost	Ophthalmology
Celecoxib	Pain
Diclofenac	Pain
Fentanyl	Pain
Gabapentin	Pain
Hydrocodone/acetaminophen	Pain
Lidocaine	Pain
Lidocaine-prilocaine	Pain
Morphine	Pain
Oxycodone	Pain
Oxycodone-acetaminophen	Pain
Oxymorphone	Pain
Tramadol	Pain
Alprazolam	Psychiatry
Aripiprazole	Psychiatry
Asenapine	Psychiatry
Buprenorphine/naloxone	Psychiatry
Bupropion	Psychiatry
Clozapine	Psychiatry
Desvenlafaxine	Psychiatry
Duloxetine	Psychiatry
Escitalopram	Psychiatry
Fluoxetine	Psychiatry
Lurasidone	Psychiatry
Mirtazapine	Psychiatry
Paliperidone	Psychiatry
Quetiapine	Psychiatry
Risperidone	Psychiatry
Sertraline	Psychiatry
Trazodone	Psychiatry
Varencicline	Psychiatry
Venlafaxine	Psychiatry
Zolpidem	Psychiatry
Albuterol	Pulmonary
Albuterol/ipratropium	Pulmonary
Alpha-1 proteinase inhibitor	Pulmonary

© 2021 Tisdale RL et al. *JAMA Network Open*.

Ambrisentan	Pulmonary
Beclomethasone	Pulmonary
Bosentan	Pulmonary
Budesonide/formoterol	Pulmonary
Fluticasone	Pulmonary
Fluticasone salmeterol	Pulmonary
Fluticasone/vilanterol	Pulmonary
Formoterol/mometasone	Pulmonary
Ipratropium	Pulmonary
Macitentan	Pulmonary
Montelukast	Pulmonary
Pirfenidone	Pulmonary
Riociguat	Pulmonary
Roflumilast	Pulmonary
Selexipag	Pulmonary
Tadalafil	Pulmonary
Tiotropium	Pulmonary
Umeclidinium	Pulmonary
Umeclidinium/vilanterol	Pulmonary
Abatacept	Rheumatology
Adalimumab	Rheumatology
Allopurinol	Rheumatology
Apremilast	Rheumatology
Certolizumab	Rheumatology
Colchicine	Rheumatology
Etanercept	Rheumatology
Febuxostat	Rheumatology
Golimumab	Rheumatology
Hydroxychloroquine	Rheumatology
Immune globulin	Rheumatology
Immune globulin	Rheumatology
Prednisolone	Rheumatology
Tofacitinib	Rheumatology
Ustekinumab	Rheumatology
Dutasteride	Urology
Fesoterodine	Urology
Finasteride	Urology
Mirabegron	Urology
Oxybutynin	Urology
Silodosin	Urology
Solifenacin	Urology
Tamsulosin	Urology

© 2021 Tisdale RL et al. *JAMA Network Open*.

Tolterodine	Urology

© 2021 Tisdale RL et al. *JAMA Network Open*.

eTable 2. Search Terms

Search Terms
ledipasvir/sofosbuvir
lenalidomide
glargine
sitagliptin
rosuvastatin
fluticasone salmeterol
pregabalin
rivaroxaban
apixaban
tiotropium
glargine
adalimumab
etanercept
sevelamer
ezetimibe
glatiramer
budesonide/formoterol
detemir
cinacalcet
aspart
esomeprazole
aripiprazole
memantine
lispro
palbociclib
ibrutinib
lidocaine
cyclosporine
sofosbuvir
dimethyl fumarate
enzalutamide
oxycodone
abiraterone
levothyroxine
liraglutide
lurasidone
imatinib
paliperidone

© 2021 Tisdale RL et al. *JAMA Network Open*.

atorvastatin
hydrocodone/acetaminophen
linagliptin
solifenacin
corticotropin
emtricitabine/tenofovir
lispro
dabigatran
canagliflozin
aspart
ambrisentan
metoprolol succinate
metformin/sitagliptin
dexlansoprazole
quetiapine
ruxolitinib
esomeprazole
efavirenz/emtricitabine/tenofovir
detemir
ranolazine
mirabegron
teriparatide
albuterol/ipratropium
duloxetine
adalimumab
gabapentin
bimatoprost
pomalidomide
rifaximin
pancrelipase
abacavir/dolutegravir/lamivudine
potassium chloride
teriflunomide
oxycodone-acetaminophen
olmesartan
fingolimod
imatinib
omeprazole
linaclotide
albuterol
pirfenidone
tamsulosin

© 2021 Tisdale RL et al. *JAMA Network Open*.

aspart protamine
daclatasvir
travoprost
darunavir
erlotinib
sofosbuvir-velpatasvir
lacosamide
fluticasone/vilanterol
nintedanib
rosuvastatin
pravastatin
raltegravir
metformin ER
nebivolol
everolimus
glargine
macitentan
albuterol
fenofibrate
dolutegravir
clobetasol
celecoxib
interferon beta-1a
immune globulin
dasatanib
colesevelam
interferon beta-1a
lispro protamine
hydroxychloroquine
nilotinib
tiotropium
bosentan
estrogen
cobicistat/elvitegravir/emtricitabine/tenofovir alafenamide
fluticasone
dronedarone
fentanyl
omega-3
ezetimibe/simvastatin
tadalafil
lubiprostone
interferon beta-1a

© 2021 Tisdale RL et al. *JAMA Network Open*.

dulaglutide
amlodipine
hydrochlorothiazide/olmesartan
ustekinumab
risperidone
lisinopril
elvitegravir/cobicistat/emtricitabine/tenofovir disoproxil
levothyroxine
saxagliptin
enoxaparin
febuxostat
divalproex
simvastatin
rivastigmine
oxycodone
morphine
brimonidine/timolol
testosterone
prasugrel
raloxifene
interferon beta-1b
diclofenac
valsartan
memantine
losartan
rivastigmine
liraglutide
rasagiline
clopidogrel
sorafenib
tolterodine
abatacept
ticagrelor
dalfampridine
formoterol/mometasone
tofacitinib
aripiprazole
dextromethorphan/quinidine
elbasvir/grazoprevir
mesalamine
human insulin isophane
desvenlafaxine

© 2021 Tisdale RL et al. *JAMA Network Open*.

atazanavir
buprenorphine/naloxone
fluticasone/salmeterol
diclofenac
brimonidine
colchicine
denosumab
sodium oxybate
riociguat
immune globulin
metformin
pantoprazole
sunitinib
umeclidinium
zoster vaccine
umeclidinium/vilanterol
epoetin
tenofovir
emtricitabine/rilpivirine hydochloride/tenofovir disoproxil
metformin/sitagliptin
exenatide
human insulin
epinephrine
apremilast
tetrabenazine
warfarin
quetiapine
certolizumab
abacavir/lamivudine
olopatadine
aspirin-dipyridamole
donepezil
pazopanib
lispro protamine
paliperidone
human insulin isophane
oxybutynin
aspart protamine
montelukast
human insulin isophane
fluticasone
diltiazem

© 2021 Tisdale RL et al. *JAMA Network Open*.

allopurinol
C1 esterase inhibitor
methotrexate
oxymorphone
estradiol
metoprolol tartrate
varenicline
alpha-1 proteinase inhibitor
budesonide
bupropion
paliperidone
collagenase
doxycycline
beclomethasone
venlafaxine
divalproex
valganciclovir
paliperidone
furosemide
prednisolone
cobicistat/darunavir
brinzolamide
escitalopram
ritonavir
roflumilast
ursodiol
eltrombopag
interferon beta-1a
chlorpromazine
ixazomib
osimertinib
sublingual fentanyl
mometasone
fesoterodine
colchicine
icatibant
ipratropium
tramadol
tetrabenazine
selexipag
latanoprost
carvedilol

© 2021 Tisdale RL et al. *JAMA Network Open*.

metformin
asenapine
mirtazapine
mesalamine
deferasirox
trazodone
niacin
golimumab
enalapril
degludec
droxidopa
silodosin
tadalafil
sertraline
dofetilide
finasteride
exenatide
difluprednate
fluoxetine
ranitidine
vancomycin
levetiracetam
tizanidine
digoxin
amlodipine/olmesartan
etravirine
fluocinonide
teduglutide
dapagliflozin
empagliflozin
alprazolam
brimonidine
lidocaine-prilocaine
estradiol
baclofen
crizotinib
clozapine
napafenac
daptomycin
isosorbide mononitrate
dutasteride
hydrochlorothiazide

© 2021 Tisdale RL et al. *JAMA Network Open*.

mesalamine
alirocumab
zolpidem
omeprazole-sodium bicarbonate
lamotrigine
nifedipine
oxycodone
valsartan-hydrochlorothiazide
metronidazole
clobazam
pancrelipase
aripiprazole
prednisone
buprenorphine
tipiracil/trifluridine
somatropin
sucralfate
dasabuvir/ombitasvir/paritaprevir/ritonavir
emtricitabine/tenofovir alafenamide
aspirin/dipyridamole

© 2021 Tisdale RL et al. *JAMA Network Open*.

eTable 3. Reasons for Exclusion from Database

Reason	Explanation/Parameters
Drug of interest (i.e. drug in top 250 by Medicare Part D spending in 2016) not included	Drugs screened by computer (see Appendix Table I. for list of search terms), then all remaining U.S.-based studies manually assessed for inclusion of drug of interest. Note that a number of combination drugs appear in the top 250 drugs; for these we specifically assessed whether that combination was studied. We did not differentiate by formulation or dose.
Study conducted with non-U.S. population and/or non-U.S. dollars	Originating outside U.S. according to Tufts CEVR database, or noted to be incorrectly tagged as conducted in U.S. population
Study conducted in pediatric population	Age in study <18 years
Drug not compared to appropriate alternative	Drug used in both arms of study, whether at same or different doses
Study not directly studying drug of interest	Primary intervention studied did not involve drug of interest, e.g. study designed to evaluate cost-effectiveness of a genetic testing intervention
Drug of interest not specifically identified	Only class of drugs explicitly mentioned, not individual drug of interest

© 2021 Tisdale RL et al. *JAMA Network Open*.
